# Safety and efficacy of mavacamten for treatment of hypertrophic cardiomyopathy: a systematic review and meta-analysis of randomized clinical trials

**DOI:** 10.1186/s43044-023-00328-7

**Published:** 2023-01-12

**Authors:** Mehrdad Rabiee Rad, Ghazal Ghasempour Dabaghi, Danial Habibi

**Affiliations:** 1 School of Medicine, Isfahan University of Medical Science, Hezarjarib Ave, Isfahan, 81746-73461 Iran; 2 Department of Biostatistics and Epidemiology, Faculty of Health, Isfahan University of Medical Science, Isfahan, Iran

**Keywords:** Hypertrophic cardiomyopathy, Mavacamten, HCM, HOCM

## Abstract

**Background:**

Mavacamten, an allosteric myosin inhibitor, is considered to be a promising drug for the treatment of hypertrophic cardiomyopathy (HCM). This meta-analysis aimed to explore the safety and efficacy of mavacamten in HCM patients.

**Main body:**

A total number of 539 patients were enrolled in four randomized clinical trials. The mean age of patients was 57.9 years and was followed for 29.3 weeks. Pooled analysis showed a significant improvement in clinical response (Log OR = 0.65; *p* = 0.01) and the number of patients with a reduction of ≥ 1 NYHA function class (Log OR = 0.64, *p* = 0.00). It was found that mavacamten did not significantly affect the Kansas City Cardiomyopathy Questionnaire (KCCQ) (SMD = 0.43, *p* = 0.08), peak oxygen uptake (PVO_2_) (SMD = 0.24, *p* = 0.42), and ejection fraction (EF) (SMD = − 0.65, *p* = 0.13) as compared with placebo. However, KCCQ (SMD = 0.65, 95% CI 0.44–0.87) and PVO_2_ (SMD = 0.49, 95% CI 0.24–0.74) improvements were statically significant in the hypertrophic obstructive cardiomyopathy subgroup (HOCM), and a significant decrease in EF (SMD = -− 1.14, 95% CI − 1.86 to − 0.42) was found in the HOCM subgroup. No significant difference was observed in the incidence rate of serious adverse events between mavacamten and placebo group (Log OR = − 0.23, *p* = 0.56).

**Conclusions:**

Mavacamten proved to be effective and well-tolerated for the treatment of HCM. Mavacamten improved the signs and symptoms of HOCM and decreased EF in these patients without serious adverse events in the clinical trials.

**Supplementary Information:**

The online version contains supplementary material available at 10.1186/s43044-023-00328-7.

## Background

Hypertrophic cardiomyopathy (HCM) is the most frequent inherited cardiovascular disorder and is responsible for most cases of sudden cardiac death (SCD) in young people, being present in 0.2% of the general population [1]. HCM is marked by an increased in left ventricular (LV) thickness ≥ 15 mm in adults, abnormal mitral valve, decreased compliance, myofibrillar disarray, and cardiac fibrosis [2, 3]. Autosomal dominant mutations in sarcomere-related genes such as cardiac β-myosin heavy chain (MYH7) or myosin-binding protein C (MYBPC3) are the leading causes for HCM [4].

There are several therapeutic options for obstructive HCM including β-blockers, the non-dihydropyridine calcium-channel blockers, diuretics, and implantable cardioverter defibrillators (ICDs) [5, 6]. However, these current agents are not fully effective and a considerable portion of patients remain symptomatic despite treatment. Besides, no method has proven successful in correcting the genetic defects. Hence, the development of a novel pharmacological approach is needed.

Mavacamten (MYC-461, camzyos) is a novel specific inhibitor of β-cardiac myosin ATPase [7]. Mavacamten decreases the number of actin-myosin cross-bridge leading to reducing hypercontractility, a mechanism involving in HCM pathogenesis [8]. Preclinical animal studies showed that use of mavacamten decreases the development of left ventricular hypertrophy, fractional shortening, and fibrosis in mice models with a mutation in the myosin heavy chain [9]. Both acute and chronic administrations of mavacamten increase left ventricular end-diastolic volume, and chronic use decreases ejection fraction in dogs [10]. Besides, treatment with mavacamten improved mitral valve anterior motion and left ventricular outflow tract (LVOT) gradient in cats from a research colony with naturally occurring HCM [11].

This promising data led to clinical experimentation and resulted in randomized controlled trials (RCTs) of mavacamten use in HCM patients. Therefore, this systematic review aimed to evaluate safety and efficacy of mavacamten in patients with HCM. A meta-analysis was performed to summarize quantitative data from previous RCTs.

## Main text

We followed the guidelines from Preferred Reporting Items for Systematic Reviews and Meta-analyses (Additional file 1: PRISMA). The search strategy was applied to Medline, Scopus, Web of Science, and the Cochrane Library for articles published from databases until May 30, 2022. The following terms were used to search MEDLINE and adapted for the other databases: ((mavacamten OR MYC-461 OR camzyos) AND ("hypertrophic cardiomyopathy" OR cardiomyopathy OR HCM OR HOCM OR "familial hypertrophic cardiomyopathy" OR "hypertrophic obstructive cardiomyopathy"). We investigated the reference lists of related studies to detect articles potentially eligible for inclusion. No language restrictions were placed.

Human studies with a diagnosis of hypertrophic cardiomyopathy included in this study if they met the following criteria: (a) double-blinded randomized clinical trials that the treatment group received placebo and the intervention group received a specified amount of mavacamten, (b) age ≥ 18 years old, (c) adequate data on clinical responses, echocardiogram parameters, Kansas City Cardiomyopathy Questionnaire (KCCQ) score, and serious adverse event at baseline and at the end-point of follow-up in both groups. Non-randomized trials, uncontrolled trials, review studies, case–control, cross-sectional, cohort studies, abstracts, and articles with insufficient data were excluded. No sample size, race, and country restrictions were imposed.

Two independent reviewers performed the literature search and checked the eligibility of each study. Collected data items included first author's last name, publication time, duration of intervention, sample size, condition of participant disease (obstructive or non-obstructive hypertrophic cardiomyopathy), mean age, dose of mavacamten used in intervention groups, and the interest outcomes including clinical response defined by a 15 mL/kg/min or greater increase in peak oxygen uptake (PVO_2_) and at least one NYHA class reduction; or a 30 mL/kg/min or greater improvement in PVO_2_ and no worsening of NYHA class, NYHA function class, PVO_2_, ejection fraction (EF), KCCQ score, and serious adverse events during the treatment.

We used the Cochrane risk of bias assessment tool (ROB2) [12] to evaluate the risk of bias of enrolled studies. The bias was assessed based on five domains: (a) randomization process, (b) intended interventions, (c) missing outcome data, (d) measurement of the outcome, and (e) selection of the reported result. Each domain of studies was classified as high, some concerns, and low risk of bias. This section was also performed by two independent reviewers. Final scores were discussed by the reviewers to make a consensus. The risk of bias for included trials is shown in Additional file 2: Appendix S1.

All analysis was performed using STATA, version 16. The odds ratio (OR) and 95% confidence intervals (CI) were calculated for dichotomous outcome indicators. The standardized mean difference (SMD) and 95% CI were calculated for continuous outcome indicators. A random-effects model was used according to between-trial heterogeneity. We assessed heterogeneity using *I*^2^ and Q statistics [13]. The heterogeneity was considered significant if values of *I*^2^ were higher than 50%. Subgroup analysis was performed to explore the possible causes of heterogeneity. *P* < 0.05 was considered statistically significant.

**Fig. 1:**
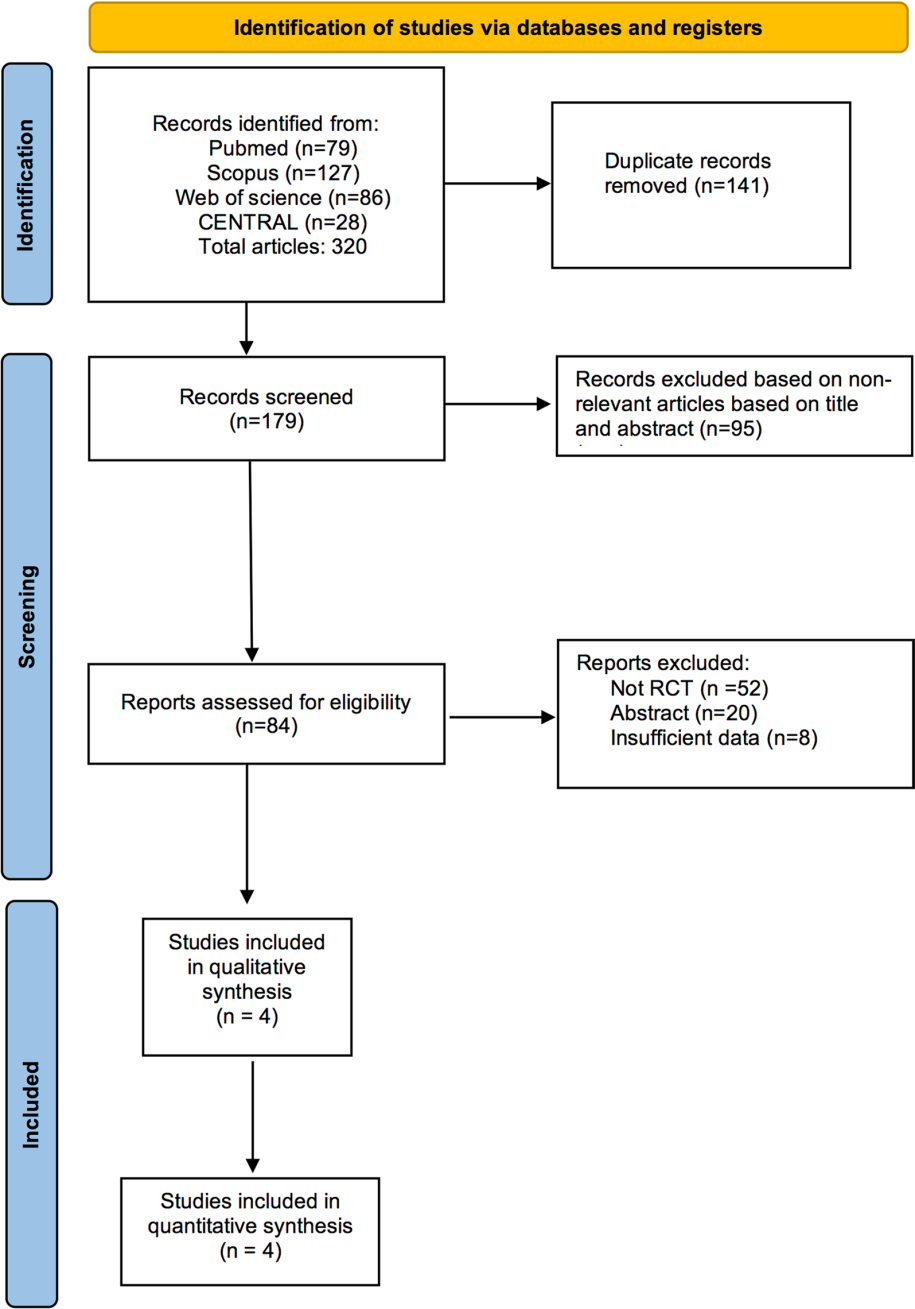
Study flow diagram based on Preferred Reporting Items for Systematic Reviews and Meta-analyses protocols (PRISMA-P) recommendation

As showed in Fig. 1, we screened the title and abstract of 320 potentially eligible studies. 236 citations were excluded since they were duplicated or non-relevant articles. Therefore, 84 publications were assessed for eligibility and four randomized clinical trial comparing safety and efficacy of mavacamten consumption with placebo met the inclusion criteria [14–17]. A total number of 539 patients were included in this meta-analysis. 278 patients were in the intervention group, and 261 patients in placebo group. The overall mean age of included participants was 57.9 years. All studies focused on the mavacamten plasma concentration which varies from 200 to 700 ng/mL. The mean duration of follow-up was 29.3 weeks. Study characteristics are mentioned in Table 1.

**Table 1:** Detailed characteristics of the included studies

Study	Year	Condition of disease	Length of treatment (weeks)	Number of participants (intervention/placebo)	Mean age	Male (%)	Dose of mavacamten
Ho et al. [14]	2020	Non-HOCM	24	40/19	53.9	42.4	200–500 ng/mL plasma concentration
Olivotto et al. [15]	2020	HOCM	30	123/128	58.5	59.3	350–700 ng/mL plasma concentration
Saberi et al. [16]	2020	HOCM	30	17/18	60.3	57.1	350–700 ng/mL plasma concentration
Spertus et al. [17]	2021	HOCM	30	98/96	57.9	60.8	350–700 ng/mL plasma concentration

HOCM, hypertrophic obstructive cardiomyopathy

**Fig. 2:**
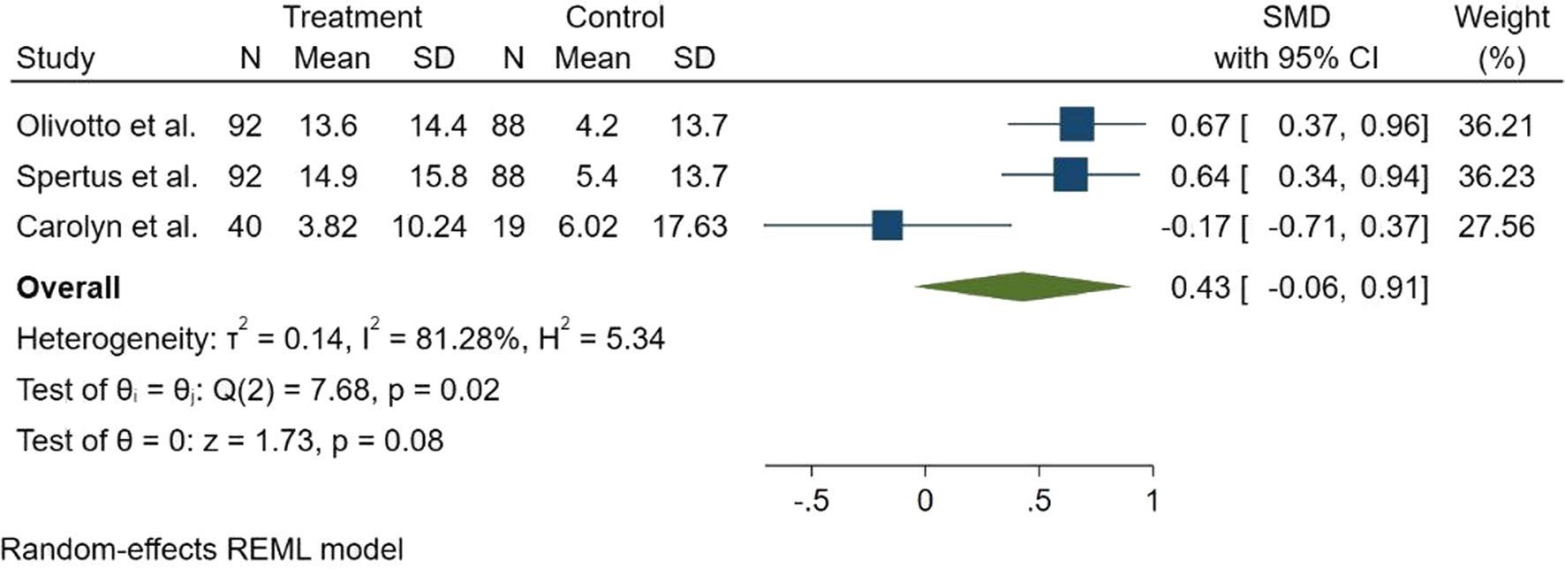
Forest plot for Kansas City Cardiomyopathy Questionnaire (KCCQ) score

KCCQ was measured in three studies. The forest plot of comparison for KCCQ between placebo and mavacamten group showed no statically significant difference (SMD = 0.43, 95% CI − 0.06 to 0.91, *p* = 0.08) and there was significantly heterogeneity between studies (*I*^2^ = 81.28%) (Fig. 2). Subgroup analysis was done. Type of HCM (HOCM or non-HOCM) was the source of heterogeneity, and KCCQ improvement was statically significant in the HOCM subgroup (SMD = 0.65, 95% CI 0.44–0.87). However, it was not statically significant in the non-HOCM group (SMD = − 0.17, 95% CI − 0.72 to 0.38).

**Fig. 3:**
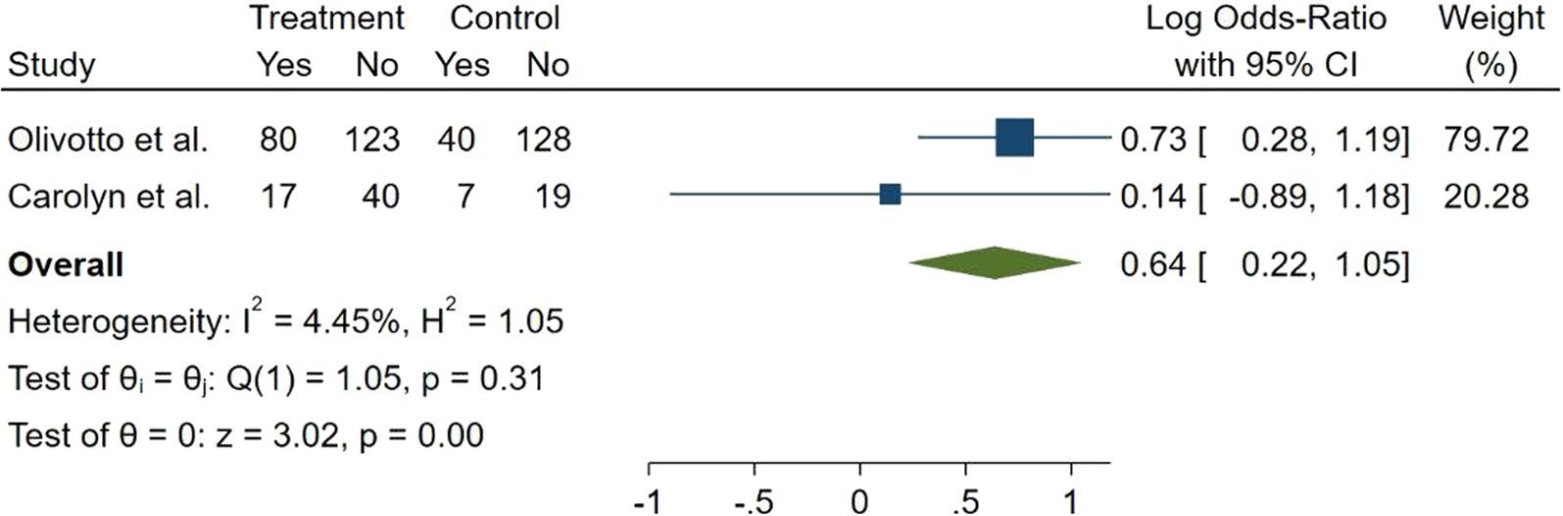
Forest plot for the number of patients with ≥ one reduction in NYHA function class

Number of patients who have ≥ 1 NYHA class improvement from the base line was measured in two studies. The forest plot of overall evaluation revealed that incidence of NYHA class improvement in mavacamten group was significantly higher than placebo group (Log OR = 0.64, 95% CI 0.22–1.05, *p* = 0.00, *I*^2^ = 4.45%) (Fig. 3).

**Fig. 4:**
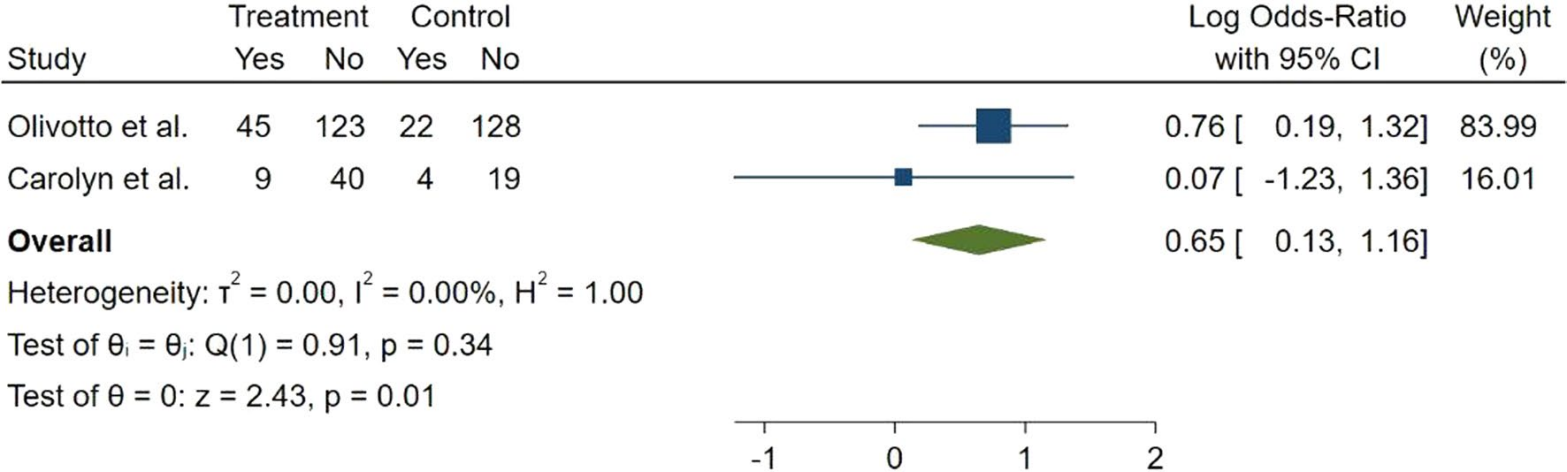
Forest plot for clinical response

Clinical response to mavacamten treatment was evaluated in two study. Clinical response considered as ≥ 1.5 ml/kg/min improvement in the PVO_2_ and ≥ 1 NYHA class reduction comparing to baseline, or ≥ 3 ml/kg/min increase in the PVO_2_ and no worsening of NYHA function class. The forest plot of overall evaluation showed that patients in mavacamten group have more chance of meeting complete clinical response compared with placebo group (Log OR = 0.65, 95% CI 0.13–1.16, *p* = 0.01, *I*^2^ = 0.00%) (Fig. 4).

**Fig. 5:**
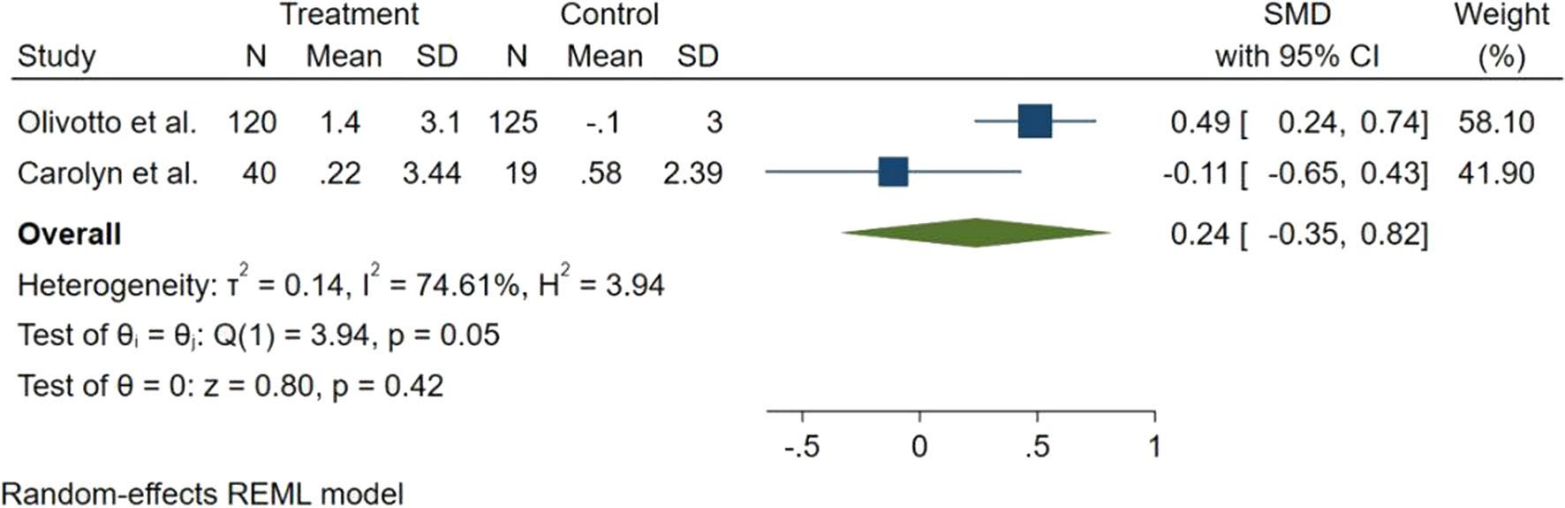
Forest plot for peak oxygen uptake (PVO_2_)

PVO_2_ evaluated in two studies. One on HOCM patients and one on non-HOCM patients. The forest plot of overall comparison of PVO_2_ revealed no statically significant change in the PVO_2_ (SMD = 0.24, 95% CI − 0.35 to 0.82, *p* = 0.42), with a significant heterogeneity (Fig. 5). Subgroup analysis was performed based on type of HCM. Source of heterogeneity was type of HCM and significant improvement of PVO_2_ was seen in the HOCM subgroup (SMD = 0.49, 95% CI 0.24–0.74), but the decrease in the non-HOCM subgroup was not statically significant (SMD = − 0.12, 95% CI − 0.76 to 0.51).

**Fig. 6:**
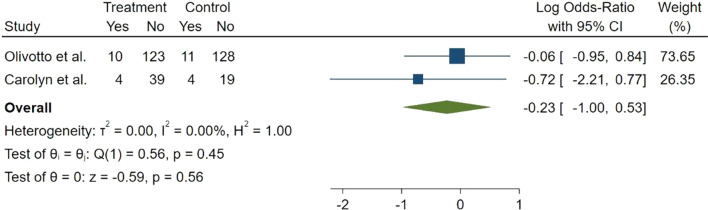
Forest plot for serious adverse events

The incidence of serious adverse events was reported in two studies. There was no significant difference in the incidence of serious adverse events between two groups (Log OR = − 0.23, 95% CI − 1.00 to 0.53, *p* = 0.56, *I*^2^ = 0.00%) (Fig. 6).

**Fig. 7:**
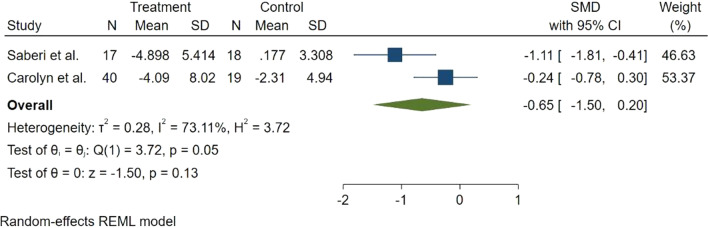
Forest plot for ejection fraction (EF)

EF was measured in two studies. One of them was on patients with HOCM, and another study was on patients with non-HOCM. The forest plot of overall comparison of EF between placebo and mavacamten suggested no statically significant change in the EF (SMD = − 0.65, 95% CI − 1.50 to 0.20, *p* = 0.13), with a significant heterogeneity between these two studies (Fig. 7). Subgroup analysis showed that the source of heterogeneity is the type of HCM. Although there was a significantly EF decrease in the HOCM subgroup (SMD = − 1.14, 95% CI − 1.86 to − 0.42), EF decrease in the non-HOCM subgroup was not statically significant (SMD = − 0.35, 95% CI − 0.80 to 0.30).

Four RCTs allocating 539 participants diagnosed with HCM were included in this study. To the best of our knowledge, this is the first RCT-based meta-analysis evaluating the efficacy of mavacamten among HCM patients. Overall, results indicated that provision of mavacamten improves NYHA function class and clinical response among patients with HCM. Results on the effect of mavacamten administration on KCCQ score and PVO_2_ were controversial. This meta-analysis showed no significant effect for mavacamten use on KCCQ score and PVO_2_ in patients with HCM. However, the subgroup analysis showed that mavacamten consumption may improve KCCQ score and slightly decrease EF in the HOCM group. Nevertheless, only one study on HCM patients measured EF changes. Therefore, the results about the EF reduction cannot be reliable due to lack of enough data. Besides, our meta-analysis revealed that tolerability outcomes of mavacamten were equal to placebo.

AHA/ACC Guideline recommended that LV wall thickness ≥ 30 mm and late gadolinium enhancement as clinical risk factors for SCD in HCM patients [18]. Moreover, several studies revealed that LVOT obstruction increases the risk of SCD in HCM patients [19–21]. Saberi et al. showed a lower maximum LV wall thickness with no significant change in late gadolinium enhancement in cardiac magnetic resonance imaging after treatment with macavamten [16]. Olivotoo et al. demonstrated that mavacamten causes more reduction in post-exercise LVOT gradient compared with placebo [15]. Therefore, it seems reasonable to offer mavacamten as a novel tool for SCD prevention.

Mavacamten has been shown to have a negative inotropic effect that is mediated by direct inhibition of cardiac myosin [7]. The results of this meta-analysis demonstrated that mavacamten administration is not associated with a significant decrease in EF in patients with HCM. However, the maximum duration of follow-up in the included studies was 30 weeks. Furthermore, the target serum concentration of mavacamten was 200–800 ng/ml. Therefore, higher doses of mavacamten may have a negative impact on EF after a longer follow-up period.

This study does have a few limitations. First, the number of RCTs included in this meta-analysis was low. More studies with a larger number of participants are needed to draw definite conclusions. Second, there was some heterogeneity between populations included in this meta-analysis. Third, we analyzed only six indicators and could not able to comprehensively evaluate other factors.

## Conclusions

In conclusion, evidence from four RCTs indicated that mavacamten could improve the clinical response and NYHA function class in patient with HCM. Besides, mavacamten administration was associated with enhanced peak VO_2_ and KCCQ score in obstructive HCM patients. Administration of mavacamten was associated with no significant serious adverse events.

## Supplementary Information

**Additional file 1.** PRISMA checklist.

**Additional file 2.** Risk of bias for included studies.

## Data Availability

Not applicable.

## Abbreviations

CI: Confidence intervals
EF: Ejection fraction
HCM: Hypertrophic cardiomyopathy
HOCM: Hypertrophic obstructive cardiomyopathy
ICD: Implantable cardioverter defibrillator
KCCQ: Kansas City Cardiomyopathy Questionnaire
LVOT: Left ventricular outflow tract
MYBPC3: Myosin-binding protein C
MYH7: β-Myosin heavy chain
NYHA: New York Heart Association
OR: Odds ratio
PVO_2_: Peak oxygen uptake
RCTs: Randomized controlled trials
SCD: Sudden cardiac death
SMD: Standardized mean difference
