# Supplementary material for: Safety and efficacy of mavacamten for treatment of hypertrophic cardiomyopathy: a systematic review and meta-analysis of randomized clinical trials
Source: Egypt Heart J. 2023 Jan 12;75:4. doi: 10.1186/s43044-023-00328-7 (PMC9837360; doi:10.1186/s43044-023-00328-7)
Supplement: Supplementary file 2 — Additional file 2. Risk of bias for included studies. [file 43044_2023_328_MOESM2_ESM.pdf]

Safety and efficacy of mavacamten for treatment of hypertrophic cardiomyopathy: A systematic review and meta-analysis of randomized clinical trials

Mehrdad Rabiee Rad[1], Ghazal Ghasempour Dabaghi[1], Danial Habibi[2]

[1]School of Medicine, Isfahan University of Medical Science, Isfahan, Iran

[2]Department of Biostatistics and Epidemiology, Faculty of Health, Isfahan University of Medical Science, Isfahan, Iran

Corresponding author:

Ghazal Ghasempour Dabaghi

Email: ghasempoorghazal@gmail.com

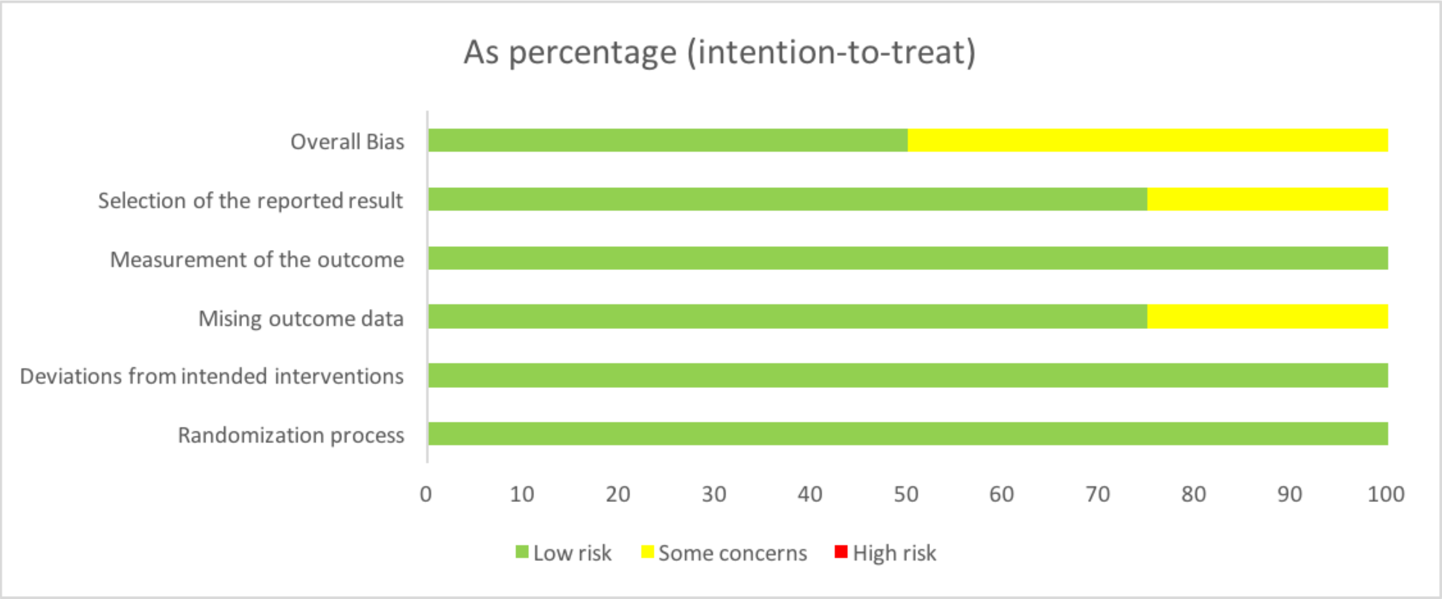
As percentage (intention-to-treat)
Overall Bias
Selection of the reported result
Measurement of the outcome
Mising outcome data
Deviations from intended interventions
Randomization process
0
10
20
30
40
50
60
70
80
90
100
Low risk
Some concerns
High risk

	Randomisation process	Intended interventions	Missing outcome data	Measurement of the outcome	Selection of the reported result	Overall
Ho et al.	+	+	+	+	!	!
Olivotto et al.	+	+	+	+	+	+
Spertus et al.	+	+	!	+	+	!
Saberi et al.	+	+	+	+	+	+
